# The Effects of Traditional Chinese Medicine-Associated Complementary and Alternative Medicine on Women with Polycystic Ovary Syndrome

**DOI:** 10.1155/2021/6619597

**Published:** 2021-02-26

**Authors:** Wenjuan Shen, Bao Jin, Yujia Pan, Yanhua Han, Tianjiao You, Zongyu Zhang, Yangfan Qu, Sha Liu, Yang Zhang

**Affiliations:** ^1^Department of Obstetrics and Gynecology, First Affiliated Hospital, Heilongjiang University of Chinese Medicine, Harbin 150040, China; ^2^Department of Obstetrics and Gynecology, Heilongjiang University of Chinese Medicine, Harbin 150040, China; ^3^Department of Internal Medicine, Heilongjiang University of Chinese Medicine, Harbin 150040, China; ^4^Department of Internal Medicine, First Affiliated Hospital, Heilongjiang University of Chinese Medicine, Harbin 150040, China

## Abstract

Polycystic ovary syndrome (PCOS) is a touchy clinical and public health problem worldwide, which adversely affects women's health and health-related comorbidities for lifetime, and represents a tremendous burden for both the family of the patient and for society. According to the diagnostic criteria used and the population studied, the prevalence rate of PCOS is between 6% and 21%. However, current conventional modern medicines for PCOS are only moderately effective at controlling the signs and symptoms, while they are not thoroughly able to prevent complications. Therefore, many patients have turned to complementary and alternative medical (CAM) treatments. CAM use is wide spread among patients with PCOS, and more than 70% of patients use CAM at one point during their diseases. The patients' primary motivations include dissatisfaction with available medications, perceive higher risk of drug side effects and crushing health burden and economic costs, desire for symptom relief, pursuit of shortening the course of disease, and the belief that CAM therapy is in accordance with the patients' values and beliefs. At present, several CAM methods have been used in women with PCOS, which has achieved obvious effects. However, biologically plausible mechanisms of the action of traditional Chinese medicine- (TCM-) associated CAM for PCOS have not been systematically reviewed. This review briefly summarizes the current progress of the impact of herbal medicine on the outcomes of PCOS and introduces the mechanisms.

## 1. Introduction

Polycystic ovary syndrome (PCOS) is one of the most common and complex endocrinopathy occurring in reproductive aged women. According to the diagnostic criteria used and the population studied, the prevalence rate of PCOS is between 6% and 21% [1]. PCOS is characterized by chronic anovulation, hyperandrogenism (HA), and metabolism disorder leading to symptoms of menstrual dysfunction, subfertility, hirsutism, acne, and obesity [2]. Women with PCOS tend to have other abnormalities, including impaired glucose tolerance, type 2 diabetes mellitus, dyslipidemia, increased prevalence of hypertension, and possibly increased risk of metabolic syndrome and cardiovascular morbidity [3]. The aetiology of PCOS remains poorly understood, but it is believed to result from a complex relationship with metabolic, endocrine, genetic, behavioral, and environmental factors. Different possible theories were reported in various studies, which included detectable insulin resistance (IR), HA, and neuroendocrine gonadotropin secretion disorders, or a combination of these factors [4]. PCOS as a whole is a touchy clinical and public health problem worldwide, which adversely affects women's health and health-related comorbidities lifetime, and represents a tremendous burden for both the family of the patient and for society. The annual cost to the National Health Service modelled the population dynamics of PCOS, healthcare costs, and quality of life for PCOS patients in the UK from 2014 to 2039 and has been estimated to be more than £237 million [5].

About 70% of people around the worldwide are interested in the prevention and treatment of diseases by complementary and alternative medicine (CAM) although there is insufficient scientific assessment evidence to prove its safety and effectiveness [6]. The National Center for Complementary and Integrative Health (NCCIH), formerly known as the National Center for Complementary and Alternative Medicine (NCCAM), defines CAM as a group of diverse medical and health-care systems, practices, and products that are not presently considered to be part of conventional modern medicine. NCCIH classifies CAM into three more types: (1) natural products (consist of herbs, vitamins, minerals, probiotics, and dietary supplements); (2) mind and body practices (include a wide variety of procedures and techniques such as yoga, acupuncture, Tai chi or qi gong, massage therapy, meditation, spinal manipulation, hypnotherapy, and relaxation techniques), and (3) other complementary health approaches [7] (other complementary health approaches cover those treatments such as traditional Chinese medicine (TCM), naturopathy, and homeopathy as well as functional medicine).

Patients with PCOS rank among the higher users of CAM. Why so many people choose CAM? Their primary motivations include dissatisfaction with available medications, perceive a higher risk of drug side effects and crushing health burden and economic costs, desire for symptom relief, pursuit of shortening the course of disease, and the belief that CAM therapy is in accordance with the patients' values and beliefs. Sometimes, the patients who resort to CAM usually use it as a “alternative” therapy, but there are moments the CAM are used to incorporate with conventional modern medicine on behalf of treating and controlling the primary disease or complications as much as possible to achieve better efficacy. Given the wide application of CAM in women with PCOS, particularly the remarkable efficacy of CAM therapy in clinical randomized controlled trials of PCOS in recent years. The purpose of this narrative review is to provide a broad overview of the types of TCM-associated CAM therapies most commonly be used for PCOS and to enable patients and practitioners who are either interested in pursuing or already employing CAM to know more about the relevant research progress and mechanism.

## 2. The Utilization of Herbal Medicine in Women with PCOS

Herbal medicine (HM) has been used in eastern Asian countries for thousands of years, and it is one of the key components of CAM. One particular form of HM, herbal formula, involves the use of some herbs in a formula to improve a set of problems or a syndrome. Herbal formula and single herb were reportedly used by over 70% of women with PCOS and are a commonly encountered condition among traditional Chinese doctors [8, 9]. Many studies showed that both herbal formula and single herb are helpful for fertility, menstrual health, and other aspects of PCOS. In view of the use of HM in PCOS are becoming popular in research and clinical practice, we summarized the outcomes of clinical and the mechanisms as follows.

### 2.1. The Utilization of Herbal Formula in Women with PCOS

#### 2.1.1. Infertility and Ovulatory Dysfunction

As the treatment of infertility has attracted much attention from PCOS patients, numerous clinical studies of herbal formula have been designed, especially, quite prevalent in China. Herbal formula had positive adjunct effects with clomiphene citrate (CC) in the management of ovulation induction for PCOS-related subfertility [10–17]. The results of Chen et al. show that the prevalence of dominant follicle, ovulation rate, and clinical pregnancy rate in the combination group was significantly superior than those in the control group (*P* < 0.05) [18]. Consistent with these data, Zhao et al. also found the same discovery in 120 subjects undergoing inducing ovulation in women with PCOS; there were significantly a higher pregnancy rate (29.0% vs 15.6%) and lower early abortion rate (12.9% vs 23.5%) in the cotreatment group than that in the CC group [19], since CC is a selective estrogen modulator known to negatively impact endometrial development. Previous studies have shown that cotreatment with herbal formula significantly increased the endometrial thickness and pregnant rate for infertility treatment of PCOS [13, 20, 21]. Furthermore, adjuvant treatment of herbal formula also plays a positive role in improving the morphology and reserve capacity of the ovary, particularly ovarian volume and basal antral follicle count (AFC) [22–26]. Ru et al. applied to CC with herbal formula or without herbal formula in 180 PCOS patients and found that endometrial thickness, ovulation function, and clinical pregnancy rate were remarkable improved (*P* < 0.05) [12]. Moreover, Danhuang Quyu capsule, Tiangui capsule, and Dingkun pill all have outstanding advantages in improving pregnancy rate and enhancing the sensitivity to ovulation-inducing drugs [13, 27]. Wei et al. carried out a prospective clinical study and reported higher single follicle rate, mature follicle rate, and clinical pregnancy rate among those subjects who received Dingkun pill and CC compared with subjects in the control group (58.0% vs. 38.0%; 98.0% vs. 78.0%; 42.0% vs. 18.0%; *P* < 0.05) [13]. Recently, letrozole has been considered as an alternative to CC and has been used in ovulating and nonovulating infertile women with PCOS, especially for patients who are against CC. With regard to cycle ovulation rate and pregnancy rate, as well as the total effective rate of intervention, the results of the present studies reveal that herbal formula conjunction with letrozole is more effective than letrozole monotherapy in the treatment of PCOS [28, 29]. In a randomized controlled trial (RCT), Yuan et al. evaluated the effectiveness and efficacy of letrozole combined with HM prescription in CC-resistant infertile women with PCOS. The data exerted a significantly higher ovulation rate, clinical pregnancy rate, endometrial thickness, and a dramatically regulated hormonal status over 6 treatment cycles in the combination group [14]. In addition, others argue that Chinese herbal formula (CHF) appears to be at least as effective as CC in ovulation induction with some potential advantages over CC [30–32]. Li in a 3-menstrual cycle human study found that CHF improved pregnancy outcome compared with CC among 120 infertile PCOS patients [30]. In agreement with Li's findings, Zhou et al. also demonstrated the benefits of CHF on ovulation rate and pregnancy rate, ovarian volume, and endometrial thickness [31].

CAM treatments are commonly used as adjunctive therapy for women when they undergo in vitro fertilization (IVF) treatment, and herbal formula is widely chosen. There are positive effects on reproductive outcomes for those PCOS patients undergoing ovulation when adjuvant treatment with CHF at different time points of IVF treatment [33, 34]. Zhang et al. randomized 98 cases of PCOS patients who would undergo in vitro fertilization and embryo transfer (IVF-ET) to the CHF group and control group; CHF group received 3 months treatment with herbal formula before IVF-ET; control group did not receive any treatment before IVF-ET; and the results presented better higher quality embryo rate, implantation rate, and pregnancy rate in the CHF group than in the control group [33]. Meanwhile, using Cangfu Congxian decoction, before 2 months of IVF-ET, could significantly increase oocyte retrieval number, high-quality embryos, and 2 PN fertilization rate [34]. Adjuvant drug of CHF could improve patients' sensitivity to medicine of superovulation when the patient received controlled ovarian hyperstimulation [35–37]. The results of Lian confirmed that, in the CHF group, patients took herbal formula until human chorionic gonadotropin (HCG) injection day and achieved a higher increment in the oocytes number and clinical pregnancy rate compared with the placebo group undergoing IVF-ET [37].

Although there are hundreds of positive results referring to herbal formulas for infertility treatment in women with PCOS, there are still negative results. Zhu et al. discovered the negative results in 54 subjects undergoing IVF-ET; they found that treatment with herbal medicine compound could not dramatically increase the numbers of fertilized oocytes, high-quality embryos, and the pregnant chance (*P* > 0.05) [38]. However, some other studies have demonstrated that although herbal formula could not change ovulation rate and pregnancy rate, herbal formula could improve the level of estradiol (E_2_), luteinizing hormone (LH), and testosterone (T) and increase the blood flow of ovary [39, 40]. The possible reasons for these different findings might be concerned with differences in the study design and compound prescription composition.

#### 2.1.2. Hormonal Status and Menstruation Cycle

With the continuous exploration of the treatment of PCOS, herbal formula has been a potential treatment for irregular menstruation and hormonal imbalance in PCOS. A pilot study conducted recently in UK found herbal formula could statistically improve menstrual rates; meanwhile, liver and kidney function and adverse events data were largely normal [17]. Additionally, as HM and Western medicine (WM) have different mechanisms of action, the combination of treatment was more effective in the regulation of menstrual cycle and hormonal status [15, 16, 24, 41]. Chen provided that Chinese medicine cycle therapy combined with metformin had beneficial effects for women with oligomenorrhea, amenorrhea, and sex hormone in PCOS [42]. Zhang conducted a randomized controlled prospective study; the results confirmed that the combination group achieved a significant reduction in T, LH, and E_2_ levels and the scores for Chinese medical syndromes (*P* < 0.05) [43]. Li and Yuan applied to an adjuvant treatment with CHF or without CHF in PCOS patients and found that hormonal serum, anti-Mullerian hormone (AMH), and Chinese medical syndromes in the adjuvant treatment group were better than those in the control group [44]. However, because the composition of Chinese herbal formulas is different, the influence on the outcomes is not completely the same. The results of Wang Ping supported the fact that, despite no significant difference in serum of E_2_, T, follicle stimulating hormone (FSH), and ovarian volume, CHF would be clinically useful, especially in patients with LH, LH/FSH, and basal AFC disturbances in PCOS [45].

Moreover, herbal formula could cause hormonal changes and modify HA with little sideeffects [46–49]. Liu et al. randomized 120 cases of PCOS-HA women to the CHF group and control group; after 3 months treatment, the results showed significant reduction in T, free androgen index (FAI), dihydrotestosterone (DHT), and dehydroepiandrosterone sulfate (DHEAS) levels and increased the level of sex hormone binding globulin (SHBG) and FSH in the CHF group than in the control group (*P* < 0.01); and CHF had better effects for modifying Rosenfield and hirsutism (*P* < 0.01), promoting recovery of menstrual and spontaneous ovulation (*P* < 0.05) [50].

Herbal formula can improve reproductive dysfunctions, but at the same time, it has beneficial effects on balancing hormone status and menstrual frequency of PCOS that is also very intriguing [15, 16, 41, 42, 51]. Susan et al. conducted a RCT of 122 patients; the results found that herbal formula adjuvant group recorded a reduction in oligomenorrhoea and other significant improvements such as pregnancy rates and quality of life compared with controls (*P* < 0.01) in these overweight patients with PCOS [52]. In one trial by Zhang et al., 291 women were identified as PCOS and underwent metformin, Diane-35, or Tiangui capsule. After 3 months intervention, women who received Tiangui capsule had a higher menstrual rate and ovulation rate and lower ovarian volume serum and T level than the metformin group; the reduction of HA was similar to who received Tiangui capsule or Diane-35. This result was similar to another result that was noted by Huang [53], who used CHF to treat PCOS women. Furthermore, the long-term effect of herbal formula was superior than that of Diane-35 after consecutive 6 months. The hirsute, acne score, and hormonal-related parameters of the herbal formula group were still significantly lower than those of the control group at the end of 6 months and 3 months of withdrawal (*P* < 0.05), while those in the control group increased.

#### 2.1.3. Metabolic Dysfunction and Emotional Disturbance

Some studies showed herbal formula displayed the similar or superior effect on ameliorating glucolipid metabolism alterations compared with WM in women with PCOS [54–56]. A randomized, double-blind, placebo-controlled trial focused on the impact of Kuntai capsule in subjects with PCOS found out a significant decrease in total cholesterol (TC), triglyceride (TG), and low-density lipoprotein level, as well as insulin and homeostasis model assessment of insulin resistance (HOMA-IR) in the CHF group than in the control group, while insulin sensitivity index (ISI) and high-density lipoprotein cholesterol (HDL-C) increased markedly. The results implied that Kuntai capsule resembled an insulin sensitizing agent in therapeutic effect [55]. The study of Wei Feng also showed that CHF was similar to metformin in improving IR [57]. The herbal formula for Bushen Quyu Huatan has marvelous effect on controlling metabolic parameters of PCOS [58, 59]. Cangfu Daotan decoction a classic prescription commonly used for the treatment of PCOS, which is effective in regulating insulin, glucose, and lipid metabolism, has no obvious adverse effects [43, 60–62]. Many studies have reported that herbal formula intensified the metabolic effects of conventional drugs and maybe a newer therapeutic option for the same purpose [26, 63–65]. During Li's study, 126 participants were advised to undergo conventional intervention, or adding HMs, there was significant improving in glycated hemoglobin (HbA1c), fasting insulin (FINS), HOMA-IR, TC, and TG parameters between the combination group and the conventional treatment group [44].

The effects of different herbal formulas on regulating abnormal metabolism are different. Some clinical trials reported controversial findings of diverse herbal formulas treatment about waist hip rate (WHR), body weight (BW) and body mass index (BMI), lipid profiles, and glucose homeostasis parameters of PCOS patients [62–66]. And some studies showed that herbal formulas in PCOS patients led to WHR reduction and BW loss [27, 66]. Administration of Tiangui capsule, Guizhi Fuling capsule, and Wenjing Shexue recipe led to a substantial decrease in BW, BMI, and other anthropometric indices [27, 39, 53], whereas the findings of Lian were not exactly the same to the above, and Lian found sequential CHF could intensify amelioration in insulin (INS) and ISI level but no changes were observed in fasting blood glucose (FBG) and WHR and BMI outcomes in IR women with PCOS [67, 68]. Fewer adverse events were found in adjuvant treatments of CHF than that in the conventional treatments [24, 43, 60, 61]. The similar discovery was found by Li, they asserted that cotherapy with Bailing capsule could conspicuously regulate metabolic parameters compared with Diane-35 alone, and the cotherapy could protect renal function with no negative effect on liver function [69]. Adjuvant therapy with herbal formula may not only help to ameliorate the glucose and lipid metabolism disorder but also to indirectly improve fertility and hormonal condition in women with PCOS [56, 70–73]. Teng suggested the optional use of herbal formula as an adjuvant therapy for infertility to modify lipid disturbances and then remarkably increase the embryo implantation rate in 227 dyslipidemia women with PCOS undergoing IVF [74].

Furthermore, PCOS is a common endocrine disorder with psychological and emotional disturbance throughout the life course of affected women. Herbal formulas appear to improve symptoms of anxiety, depression, and quality of life in PCOS patients and may enhance the overall effects [15, 75, 76]. Fu conducted a randomized controlled prospective study, which also confirmed this point; the results confirmed that the herbal formula decreased the anxiety and depression scale scores (self-rating anxiety scale and self-rating depression scale) and improved the hormonal status causing considerable alterations in ovulation and pregnancy rate [77]. We have listed some clinical trials in Table 1.

#### 2.1.4. The Mechanism of Herbal Formula Action in Women with PCOS

Regarding the potential mechanisms of herbal formula effect on PCOS, the following points deserve careful consideration. The first point is that herbal formula can be effective to improve IR and glucolipid disorders. Herbal formula improving IR is related to interventions on all fronts of the insulin signaling pathway mainly including insulin receptor, phosphoinositide 3-kinase (PI3K)/protein kinase B (Akt), glucose transporter, glycogen synthase kinase 3, mitogen-activated protein kinase (MAPK), and AMP-activated protein kinase (AMPK) as revealed by both in vitro and in vivo studies [37, 43, 44, 78–83]. Considerable herbal formulas, for instance, Cangfu Daotan decoction, Liuwei Dihuang pill, and Heqi san, result in promoted insulin sensitivity through modulation of diverse physiological and cell signaling pathways [78, 80, 82]. The effects of herbal formula are not only confined to boosting insulin sensitivity but also can be beneficial for alleviating lipid abnormality. The previous studies suggested that CHF could significantly change in glycemic index, insulin sensitivity, TG, and HDL-C, exerting protective effects against metabolic disorder [55, 64, 84, 85]. Visfatin (VF), leptin (LEP), and adiponectin (APN) are closely related to obesity [86, 87]. The imbalance of adipose cytokines such as LEP and APN secreted by adipose tissue in obese patients will also aggravate the IR of PCOS. Some experts have found that herbal formulas also can decrease serum levels of VF and leptin LEP while increase APN level, improving glucolipid metabolism and wellbeing [71, 88].

The second point is that herbal formula can enhance the endometrial receptivity (ER), which improves fertility. The major target organ of estrogen and progesterone (P) is endometrium. The estrogen and P supplied by the ovary can facilitate the proliferation and differentiation of endometrial cells, strengthening the ability of endometrium to accept embryos. Studies shown CHF can increase the levels of estrogen and P and the expressions of their receptors [13, 21, 89]. Endometrial thickness and endometrial morphology are also important parameters affecting the success of embryo implantation. Many mechanisms have verified that the defective condition of ER can be improved by regulating endometrial thickness and endometrial morphology, when undergoing treatment with herbal formula. At the same time, herbal formula treatments also are beneficial to reduce the volume of ovary and the number of ovarian cysts [12, 44, 45, 89].

Du reported that herbal formula therapy had positive effective in endometrial thickness, type A endometrium, and menstruation recovery of infertility patient with PCOS and further found higher pregnancy rates (52.63% versus 28.07%) in the combination group than conventional pharmacological therapy [90]. With endometrial microcirculation disturbance, the implantation rate is low. Herbal formula can modify the endometrial microcirculation-related indicators such as resistance index and hemodynamic index and increase endometrial blood supply, and as a result, the endometrial receptivity was improved to provide good environment for embryo implantation and improve the pregnancy rate [71, 88]. Notably, kidney herbs nourish could increase endometrial growth by promoting circulation of blood. In addition, both clinical trials and animal experiments have been certified that HF could regulate the expression of molecular biologicals, such as integrin (*α*v*β*3), vascular endothelial growth factor (VEGF), and uncoupling protein 2 (UCP2), which are closely related to ER [21, 91, 92]. The previous study showed that using herbal formula as an adjuvant therapy has remarkable improvement effect on pulsatility index (PI) and resistance index (RI), endometrial thickness, and the expression of HOXA10 in endometria while further found higher pregnancy rates (42.5% versus 22.5%; *P* < 0.05) in the combination group compared with the control group [93]. And the animal experiment also verified these benefits that herbal formula could facilitate embryo implantation and litter size by promoting endometrium and the expression of HOXA10 [94].

The third point is that herbal formula may modulate the secretion of gonadotropin and sexual hormone via hypothalamus-pituitary ovary axis and is subsequently related to the promotion of follicular maturation and the success of ovulation [45, 50, 95]. PCOS increases the gonadotropin-releasing hormone (GnRH) pulse rate, raises the LH and lowers the FSH, and elevates LH/FSH ratio which aggravates the secretion of androgen and restrains the form of dominant follicle, thus resulting in the state of PCOM and impairing ovulation. Some HF ameliorated sex hormones by markedly upregulating FSH level and downregulating LH and T levels as well as improving circulating E_2_ concentration, which could promote follicular development and induce ovulation in PCOS rats [96]. The ovarian hormones estrogen, P, and T play the role in the menstrual cycle and reproductive function. Some clinical researches have exhibited that herbal formula has beneficial effects on improving menstrual cyclicity and increasing rate of ovulation by affecting plasma levels of LH, FSH, E_2_, P, and T [24, 41, 42, 96]. Research evidences also have shown herbal formula application positively affecting the ovarian architecture by increasing the number of corpus luteum and decreasing cystic and atretic follicles [97, 98].

The fourth point is that herbal formula may exhibit better antiandrogenic effect by regulating steroidogenic enzymes and steroid receptors and gonadotropin receptors. Ovary is the major source of androgen biosynthesis. In PCOS, theca cells or granule cells overexpress mRNA encoding enzymes involved in steroidogenesis, including steroidogenic acute regulatory protein (StAR), CYP11, CYP17, and CYP19, while their overexpression could cause ovarian hormone synthesis disturbance. Studies have reported that herbal formula can reinstate the balance of androgen and estrogen by rescuing the suppressed expression of LHR, FSHR, and aromatase, thus leading to improved serum E_2_ and T concentration, the changes in cystic morphologic of ovaries, and the attenuation of the disordered estrous cycle [96, 99–102]. Many CHFs play important roles on ovarian function by regulating hormone synthesis, which regulated the expression of enzymes involved in androgen synthesis, and these changes paralleled the changes in hormone level both in vivo and in vitro [99, 103, 104]. Animal studies have confirmed that Liuwei Dihuang pill and Mahuang Tang both can promote follicular development and induce ovulation and improve the ovarian polycystic pathogenesis by modulating the dysregulation of steroid hormones and steroidogenic enzymes [80, 96]. Moreover, some researchers considered that herbal formula may play a vital function in treatment by regulating some useful cytokines' expression, such as insulin-like growth factor (IGF), VEGF, tumor necrosis factor (TNF), and inflammatory cytokines expression, thereby alleviating the symptoms of PCOS [91, 92, 105].

The last point is that herbal formula can alleviate emotional distress by relieving chronic stress, which is in connection with activity of sympathetic nervous system. Adverse mental and psychological state can affect the activity of sympathetic nervous system and disturb the release of noradrenaline (NE) and nerve growth factor, thereby affecting follicular development. Recent studies suggested that adjuvant herbal formula could assist PCOS patient better, handle psychological and mood states, as well as increase the success rate of pregnancy when they received infertility treatment [15, 77]. Sun et al. found follicle development abnormality, endocrine disturbance, increased NE level, and activation of locus coeruleus in PCOS model rat, and suggested that the beneficial role of Xiaoyaosan was correlated with the regulation of the sympathetic nerve activity [106].

### 2.2. The Utilization of Single Herb in Women with PCOS

#### 2.2.1. The Effects of Single Herb for PCOS

Single herbs use in PCOS is increasing worldwide as RCT evidence is emerging. Simple composition having no major adverse effects has made single herb medicine treatment a valuable option. Recent studies provided growing evidence that single herb medicine as CAM can help to improve health outcome and can manage the complications in women with PCOS. Many studies have revealed that single herb medicine may be a promising potent therapy in the treatment of clinical and laboratory symptoms of PCOS, including infertility, menstrual cyclicity, hormonal irregularities, IR, dyslipidemia, and anthropometric indices [102, 107]. Some single herbs (e.g., *Salvia officinalis*, *Cimicifuga racemosa*, and *Coptidis rhizome*) combined with conventional ovulation induction drugs (e.g., letrozole and clomiphene citrate) have many superiorities, such as ameliorated ovulation, thickened uterine wall thickness, increased pregnancy rates and live birth rate, as well as regulated menstruation [84, 108–111]. Two clinical trials, in China, both found that treatment with tanshinone (*Salvia officinalis*) before promoting ovulation could increase ovulation and pregnant rate [112, 113]. *Cimicifuga racemosa* might affect infertility for PCOS not only using alone but also in combination with other medications [109, 110, 113]. *Cimicifuga racemosa* plus CC also could increase clinical pregnancy rates [109, 110]. Additionally, *Cimicifuga racemosa* used alone resulted in a significant reduction in the LH level and LH/FSH ratio as well as increase in endometrial thickness, which had similar advantages to CC in the regulation of ovulation induction [109]. Some studies have found that BBR can not only improve metabolism but also has a positive effect on reproductive endocrine and reproductive outcome in patients with PCOS [114, 115]. Therefore, single Chinese herb may be popularized as a new inducing agent with good ovulatory rates and fewer sideeffects. There are still some controversies about the benefits from BBR (*Coptidis rhizome*) on fertility of PCOS. Several researches pointed out that BBR, as an assisted drug, has a positive effect on ovulation or pregnancy outcome [116]. However, a RCT showed adding BBR to letrozole did not promote fecundity in PCOS [117]. Single herb medicines also play a remarkable role in balancing menstrual pattern and the hormone status [110, 118–121]. Kort et al. investigated the effect of cinnamon on menstrual cyclicity in PCOS. After 6 months of intervention, menstrual cyclicity and menstrual flow significantly improved in cinnamon group, whereas parameters in the placebo group did not [118]. *Vitex agnus* was found to have the same and even better effect to oral contraceptives in menstrual regularity for PCOS with less adverse effects [122, 123]. It seems that fenugreek seeds are effective in the regulation of menstrual cyclicity and had promising effects on fertility [111].

Curcuma, Pueraria, and cinnamon have been explored to have strong anti-hyperinsulinemia, anti-hyperlipidemia, and antioxidant properties. Besides adjusted oxidative stress, Curcuma had remarkable decreased effects on BMI, FBG, serum insulin, TC, and low-density lipoprotein cholesterol (LDL-C) [84, 124, 125]. Using Curcuma along with metformin could cause a significant reduction in WHR and HOMA-IR in addition to a remarkable advance in glucose disposal rate (GDR) [84, 85, 125]. Several surveys of *Salvia officinalis* and Pueraria demonstrated their efficacy in the regulation of metabolic parameters and menstrual cycles [126–130]. Amini et al. also showed that the consumption of *Salvia officinalis* extract could lead to a statistically decrease in BW, waist circumference (WC), and diastolic blood pressure level [130]. The results also showed significant improvements in glycemic control and insulin sensitivity due to the levels of insulin, FBG and HOMA-IR were markedly decreased, and the quantitative insulin sensitivity check index (QUICKI) was observably increased in the *Salvia officinalis* group. A survey reported the advantageous impact of Pueraria on normalizing the menstrual cycle, IR markers, and serum level of triglyceride when used for 12 weeks [131]. Cinnamon and Curcuma have similar therapeutic effects on glucolipid profile, such as reducing FBG, IR, TC, and TG and enhancing HDL-C level [132–134]. However, there still are different opposite opinions, and some experts suggest that administration of cinnamon may not improve IR and serum TG level [118, 134]. The inconsistent effect of single herb treatment for PCOS may due to the differences in sample size, duration of use, dosage, and different drug forms. We have listed some studies' results in Table 2.

#### 2.2.2. The Mechanisms of Single Herbs for PCOS

The main mechanisms of the effectiveness of single herbs (extract) in PCOS are not yet fully understood. Nevertheless, the following points deserve careful consideration. The first point is that single herb may improve the menstrual cycle, ovulation, and fertility by regulating the secretion of endocrine hormones. Single herb may act directly on the hypothalamus to affect the hypothalamo-pituitary axis and reduce the release of gonadotrophin-releasing hormone, therefore improving the hormonal balance of LH, FSH, and T [133, 135, 136]. Reduction of the LH level increases the sensitivity of ovarian tissue to circulating FSH, contributing to alleviation of the symptoms of excessive androgens, the follicular growth, and ovulation. Also, single herbs have estrogen-like function, which was directly related to improve the implantation rate and pregnancy outcome, and can decrease the serum level of androgens by increasing the level of SHBG [137]. It has been proved that *Vitex agnus* could regulate the menstrual frequency and enhance fertility capacity by inducing the growth in the level of midluteal P, normalizing FSH and LH release, as well as inhibition of type II dopamine receptors [138]. The single herb may have agonistic and/or antagonistic effects on different oestrogenic receptors.

The second point is that single herb can reduce the IR and improve glucose homeostasis parameters of PCOS by affecting the insulin signaling pathways, which manage insulin-stimulated glucose uptake and glycogen synthesis [139, 140]. The animal studies have displayed that the extract isolated from the single herb, including flavonoids, polyphenols, diterpenoids, and triterpenoids, can potentiate glycogen synthesis increase, stimulate glucose uptake, and increase insulin sensitivity by activating insulin receptors and stimulating autophosphorylation of the insulin receptors [139, 141]. Polyphenol polymers, for instance, can exert better hypoglycemic effects by reinforcing insulin signaling at the postreceptor level and stimulating PI3K activity, and consequently, the activation of this pathway could cause the translocation of glucose transporter (GLUT) receptors, finally advancing glucose utilization by facilitating intracellular glucose transport and increasing glycogen synthesis.

The third point is the hypoglycemic effect of single herb may be related with stimulation of insulin synthesis and secretion from the beta-pancreatic cells of Langerhans [142]. There also is a cognition that single herb could affect the activity of digestive enzymes, such as pancreatic and intestinal lipase, alpha-amylase, and pyruvate kinase, which are involved in the digestion and absorption of glucose and lipid [143]. Lastly, the single herb may modulate the expression of genes closely related to cellular glucose absorption and metabolism [144].

## 3. The Utilization of Acupuncture Combined with Herbal Medicine

Acupuncture involves the insertion and retaining of very fine needles into specific anatomical points and has been used in eastern Asian countries to regulate the women's health disorders for many centuries. The major theory of acupuncture is based on Chinese medical theory which believes that there is a kind of energy flow through the body; when the energy flow is represented as a balance of Yin and Yang or Qi and Xue, the body is disease-free. In view of the high security of acupuncture treatment, transient adverse effects including pain, bleeding, redness, and hematomas are uncommon. Acupuncture therapy is now accepted, worldwide, as a kind of therapy of CAM. As early as two 2500 years ago, doctors are often used in combination with acupuncture and Chinese medicinal herbs together to treat gynaecological diseases in China. The Handbook of Prescription for Emergencies describe that “Those who apply acupuncture without medicine or medicine without acupuncture are especially not good doctors. Those who know both medicine and acupuncture are good doctors.” More importantly, herbal medicine combined with acupuncture has advantages over each alone.

A RCT trial was delivered in hospital of China in oligomenorrhea women with PCOS, and 88 patients were randomized (1 : 1) to abdominal acupuncture-medicine group or abdominal acupuncture for 3 months. During the 3 months of intervention, menstrual cycles were more frequent and menstrual blood volume was more increased in patients receiving acupuncture and Bushen Huoxue decoction compared with patients receiving acupuncture (*P* < 0.05) [145]. Acupuncture therapy adjuvant to HM could affect reproductive endocrinology and medium- to long-term functional outcome [146, 147]. Yin et al. not only found statistically significant improvements in menstrual rates but also found the BW and AMH were significantly lower; however, the ovulation and pregnancy rates were higher in the acupuncture-herbal medicine group than the control group in 120 infertility women with PCOS [147].

Electroacupuncture (EA) is considered to be an effective alternative to conventional needle acupuncture, is valuable in patients with PCOS, is a therapy where fine needles are placed in the skin and underly muscle tissue at specific areas of the body, and then input current flow in the near human body bioelectricity. Yu and Liao randomized 67 cases of obese patients of PCOS to the combination group and control group, combination group was treated with EA and Tiankui capsule, and control group was only treated with EA [148]. Results showed that BMI, WHR, BW, and FINS decreased and ISI and APN were higher in the combination group than that in the control group (*P* < 0.01). Su et al. also found the similar results; moreover, there were significantly improvement of LH, LH/FSH, T, and leptin in this study [149]. The combination treatment could better alleviate the symptoms and endocrine indices, insulin sensitivity, leptin, and APN levels, to improve the quality of life of women with PCOS. Overall, in recent ten years, many RCTs have proved that the combination of acupuncture and herbal medicine has positive impact on reproductive dysfunction, endocrine disorder, and abnormal glucolipid metabolism in PCOS [145, 146, 148–150]. The possible mechanisms of action of acupuncture for PCOS are as follows: (i) lower high sympathetic nerve activity and regulate parasympathetic and sympathetic activity; (ii) regulate the central nervous system through the hypothalamus-pituitary-ovary (HPO) axis and hypothalamus-pituitary-adrenal (HPA) axis; (iii) modulate the metabolic system by modulating expression of genes related to IR, obesity, and sympathetic activity in skeletal muscle and adipose tissue [151–153]. The animal study confirmed that combination of acupuncture and medicinal herb significantly enhanced curative effects by improving the absorption of salvianolic acid B which were extracted from the Chinese medicine formula in the PCOS rat mode [154]. The research of Yu revealed that the mechanism of combination would be EA plus herbal medicine can restore the equilibrium between Yin and Yang, as well as Qi and blood; recover kidney function, the chong and conception vessels, and the uterus; and improve endocrine function and ovarian microcirculation [148].

## 4. Dietetic Therapy and Its Mechanism of Action in PCOS

Dietetic therapy is one of the key components of TCM, according to traditional theory of TCM, and food is considered as medication. As food and medication are the same in terms of nature, origin, taste, and function, they are equally important in preventing and curing diseases [155]. Moreover, food is considered as tonic, which can treat various deficiencies including “Yin,” “Yang,” “Qi,” and “Xue,” and it can help individuals smooth body mechanisms and facilitate rehabilitation. Dietetic therapy insists that different foods have different effects on “Yin” and “Yang,” “Qi” and “Xue,” and “Zang Fu” organs. Fitting dietetic therapy can nourish “Zang Fu” organs and promote the rehabilitation; oppositely, unfitting diet will lead to the imbalance of “Yin Yang” and “Qi Xue,” even the unhealthy condition of “Zang Fu” organs. Dietetic therapy has played an important role in auxiliary treatment for PCOS. Tea, soup, and porridge are most common forms of TCM dietetic therapy. Dietetic therapies are quite suitable for PCOS patients, particularly in women who intend to be conceptive. Both meat soup and rice porridge add some herbals such as Radix Astragali Seu Hedysari, Rhizoma Polygonati Odorati, Rhizoma Dioscoreae, and Radix Ophiopogonis, to promote microcirculation and reproductive endocrine function of the ovary [156, 157].

All these foods have therapeutic effects, and these are what the doctor provided as therapy to patient, according to individual's health condition and physical problems. The food can nourish kidney, strengthen spleen, regulate Qi flow, and generate blood. Modern pharmacological research discovers that the adjunct ingredients Radix Astragali Seu Hedysari, Radix Ginseng, and Radix Ophiopogonis contain polysaccharide fractions which have antioxidant, antidiabetic, hypolipidemic, and immunomodulatory activities [158]. Many herbs, meanwhile, are rich in diosgenin which contains some synthetic materials necessary for steroid hormones, and these herbs have a similar effect as sex hormones and can promote gonadal. Recently, tea as a natural herbal medicine also has been widely considered in treating PCOS and has been intensely researched. Green tea, a commonly consumed beverage in Asia, has been found to exert beneficial effects on the endocrine system, on glucose and lipid metabolic. The latest findings of Tehrani et al. show that green tea consumption has a significant effect on overweight and obese women with PCOS, leading to weight loss and a drop in fasting insulin and free testosterone level [159]. Other studies also found that the weight, BMI, waist, and hip circumference in the green tea group were markedly declined [160, 161]. In a prospective, double-blind, placebo-controlled RCT, Grant found that spearmint herbal tea has significant antiandrogen effects, and it may be a natural and helpful treatment for hirsutism in PCOS [162]. Marjoram tea showed beneficial effects on the hormonal indicator of PCOS women, improving insulin sensitivity and reducing the levels of adrenal androgens [163]. However, when the doses of green tea were inadequate, different study had different results about glucose and lipid profile [164]. We have listed some clinical trials in Table 3.

In general, the major potential mechanisms in the support of PCOS women's health by tea involve the following [165, 166]: (i) antioxidant properties, anti-hyperlipidemic, and anti-diabetic activity; (ii) reducing carbohydrate absorption by inhibition of various digestive enzymes; (iii) increased glucose uptake in skeletal muscle, while decreased glucose uptake in adipose tissue.

## 5. Moxibustion on Patients with PCOS

Moxibustion is a noninvasive CAM therapy which has the advantage of good clinical efficacy and no toxic effects. It is characterized by the use of moxa as burning material directly or indirectly at acupoints. TCM theory considers that moxibustion therapy can not only dredge meridians and regulate qi-blood but also has a dual effect of tonification and purgation. It has been used to prevent and cure various diseases for more than 2000 years. Recent clinical and animal studies have testified that moxibustion therapy could alleviate the symptoms and/or pathology of PCOS.

In the clinical setting, moxibustion has been used to combine with oral herbal medicine (OHM) or WM in treating PCOS, especially in East Asia. Combination treatment of moxibustion is beneficial to enhance the therapeutic effect of infertility in PCOS. Both moxibustion plus OHM and moxibustion combined with OHM plus WM can get significantly a higher ovulation rate and pregnant rate than that of WM alone [167–170]. In a clinical trial, 187 infertility women with PCOS were randomized to receive CC, CC plus moxibustion, or CC plus HCG. Combination group of moxibustion had more dominant follicles compared to the CC group (*P* < 0.05) and achieves a similar effect of ovulation induction compared to the HCG group (*P* > 0.05) [171]. Findings implicated that moxibustion treatment can improve the therapeutic effects of conventional WMs including letrozole, clomiphene citrate, oral contraceptives, and metformin [167–169, 172]. With regard to the parameters of reproductive hormone, the combination group of moxibustion was associated with significantly lower levels of LH, FSH, and T [169, 170, 173]. Furthermore, adjuvant treatment of moxibustion offers advantages over WM alone in the improvement of efficacy and safety of drugs, significantly reducing the occurrence of luteinized unruptured follicle syndrome (LUFS) [171, 174]. Hence, moxibustion combined with OHM, which could normalize the sex hormones, may be useful for the PCOS women who plan to get pregnant. The therapeutic effect of moxibustion may be related with a series of physiological responses to heat stimulation generated by burning moxa from patients' and chemical stimulation from the pharmaceutical components of moxa [175]. Pharmacological research displays that the moxa, which is rich in flavonoids and polysaccharides, has strong antioxidant activity. Modern scientific research found that moxibustion exerted significantly anti-inflammatory effect, and it also can ameliorate the body immunity by regulating immune factors and immune cells [176–178]. Moxibustion on governor vessel can effectively adjust the biased state of constitution of people with yang deficiency constitution and observably elevates the levels of immunoglobulin M (IgM), immunoglobulin G (IgG), serum supple C3, and serum supple C4 [179, 180]. These are the potential mechanisms of moxibustion therapy.

## 6. Summary

At present, the majority of conventional western drugs contain single active ingredients which are active against a single biological target. However, because of the complexity of the human body, the western drug treatment might seem rather simplistic and limited for PCOS. And then, more and more women with PCOS turn to CAM to treat ovulatory and menstrual dysfunction, hormonal imbalance, insulin resistance, and other mental and psychological problems. Among the four kinds of complementary and alternative therapies for PCOS discussed in this review, herbal formula and single herbal are the most commonly used main therapies of PCOS. Moreover, there are combination therapies of acupuncture and herbal medicine, dietetic therapies, and moxibustion therapies which were used for the treatment of PCOS. All of these therapies of CAM can contribute to improve the symptoms of PCOS women in different degrees. A great deal of RCT data are available for HM; however, owing to the lack of blinding in most of the studies, it is likely that the results have a tendency to bias. High-quality designs are desperately needed to assess the efficacy of herbal medicine-associated CAM for PCOS. Additionally, novel and innovative therapies of CAM such as combinational methods are needed in treating PCOS. Importantly, herbal medicine-associated CAM therapy as a promising therapy for the patients with PCOS is worthy of further research in the near future.

## Figures and Tables

**Table 1:** Summary of randomized studies of the effect of herbal formula on PCOS outcomes.

Authors (year)	Sample size	Composition	Interventions	Duration of study	Results	AEs	References
Mo et al., 2019	148	Bushen Quyu Huatan decoction	Treatment arm: CHF + clomiphene citrate Control arm: clomiphene citrate capsules	3 cycles	Cure (18) Markedly improved (30) Moderately improved (9) Ineffective (17) Overall efficacy: 77.02% Pregnancy rate level increased; PSV and PI levels of ovarian artery increased; RI level of ovarian artery decreased; LH, LH/FSH, and T levels decreased	Nausea (T: 3, C: 1); abdominal distension (T: 3, C: 2); stomach ache (T: 2, C: 3); blurred vision (T: 1, C: 1); no SAE	[10]
Luo et al., 2018	100	Yangyin Shugan capsule	Treatment arm: CHF + clomiphene citrate Control arm: clomiphene citrate	3 menstrual cycles	Cure (12) Markedly improved (20) Moderately improved (6) Ineffective (12) Overall efficacy: 76.00% Pregnancy rate level increased; FSH and E_2_ levels increased; LH level decreased; PI and RI levels increased	Nausea and vomiting (T: 2, C: 0); diarrhea (T: 1, C: 0); abdominal distension (T: 0, C: 4); stomach ache (T: 0, C: 2); blurred vision (C: 1); no SAE	[11]
Ru et al., 2013	180	Kidney-supplementing blood-quickening liver-clearing formula	Treatment arm: CHF + clomiphene Control arm: clomiphene	3 menstrual cycles	Cure (61) Moderately improved (22) Ineffective (7) Overall efficacy: 92.2% Ovulation rate and pregnancy rate levels increased; endometrial thickness level increased; LH and T levels decreased; FSH level increased	NS	[12]
Chen et al., 2017	150	Zuogui soothing liver decoction	Treatment arm: CHF + clomiphene Control arm: clomiphene	6 months	Cure (44) Markedly improved (3) Moderately improved (21) Ineffective (7) Overall efficacy: 90.67% Ovulation rate, pregnancy rate, and dominant follicular rate levels increased; LH, FSH, and T levels decreased	Intrauterine membrane thinning (T: 6, C: 8); OHSS (T: 2, C: 3); no SAE	[18]
Ding et al., 2014	355	Cangfu Daotan decoction	Treatment I arm: clomiphene + HMG + HCG Treatment II arm: CHF + clomiphene + HMG + HCG Control arm: no intervention	3 cycles	Pregnancy rate level increased; endometrial thickness level increased; PI and RI levels decreased; HOMA-IR level decreased; UCP2 level increased (treatment II)	NS	[21]
Wang et al., 2016	110	Bailing Tiaogan decoction	Treatment arm: CHF + ethinylestradiol cyproterone acetate + clomiphene Control arm: ethinylestradiol cyprogesterone + clomiphene	4 menstrual cycles	Pregnancy rate and endometrial thickness levels increased; ovarian volume level decreased; TCM symptom scores level decreased; FFA and CRP levels decreased; *β*-EP level increased	NS	[22]
Zhang et al., 2014	291	Tiangui capsule	A arm: Tiangui capsule B arm: metformin C arm: Diane-35	3 months	Markedly improved (47) Moderately improved (35) Ineffective (28) Overall efficacy: 74.55% HOMA-IR level decreased; weight level decreased; TC level decreased; FSH and T levels decreased; ovary volume level decreased	Mild in B arm: numbness, nausea, diarrhea; mild in C arm: the lower limbs are swollen and the weight gain is obvious; no SAE	[27]
Li et al., 2017	120	Bushen Huoxue Cupailuan decoction	Treatment arm: CHF Control arm: clomiphene citrate	3–6 menstrual cycles	Pregnancy rate level increased; the number of follicles, endometrial thickness, and ovarian volume levels increased; INS level decreased; FSH and E_2_ levels increased; LH, PRL, and T levels decreased	NS	[30]
Yu et al., 2019	175	Yushi Qinggan recipe	Treatment arm: CHF Control arm: Diane-35	3 months	Cure (12) Markedly improved (16) Moderately improved (28) Ineffective (18) Overall efficacy: 75.68% Cycle ovulation rate and pregnancy rate levels increased; 0.5hINS, 1hINS_,_ 2hINS_,_ and IAUC levels decreased; LH, LH/FSH, T, FT, SHBG, and DHEAS levels decreased; endometrial thickness, blood flow of maximum uterine artery velocity increased	Irritable and nausea (T: 0, C: 2)	[32]
Zhang, 2011	98	Invigorating kidney and removing phlegm fang	Treatment arm: CHF Control arm: no intervention	3 months	Pregnancy rate, high-quality embryo rate, and embryo implantation rate levels increased; FINS level decreased; T and E_2_ levels decreased	OHSS (T: 3, C: 6); no SAE	[33]
Qian et al., 2015	120	Tonifying kidney and removing blood stasis decoction	Treatment arm: CHF + clomiphene Control arm: clomiphene	6 menstrual cycles	Cure (31) Moderately improved (20) Ineffective (9) Overall efficacy: 85.0% Ovulation rate and pregnancy rate levels increased; BBT double-phase rate level increased; LH, FSH, and LH/FSH levels decreased	AEs: none	[41]
Zhang, 2015	90	Qingre Yangyin recipe	Chinese herbs arm: CHF Western medicine arm: metformin Combined arm: CHF + metformin	3 months	BBT double-phase rate level increased; BMI level decreased; FPG, 2hGLU, FINS, 2hINS, HOMA-IR, and leptin levels decreased; APN level increased; LH, PRL, T, and E_2_ levels decreased (combined arm)	NS	[43]
Liu et al., 2018	120	Dihuang wan combined with Xianggui Erchen tang	Treatment arm: CHF Control arm: ethinylestradiol and cyproterone acetate	3 menstrual cycles	Markedly improved (33) Moderately improved (22) Ineffective (5) Overall efficacy: 76.67% Menstruation recovery rate, ovulation recovery rate, and BBT double-phase rate levels increased; BMI and WHR levels decreased; IGF-1, LP, and HOMA-IR levels decreased; T, DHT, DHEAS, FAI, LH, LH/FSH, and PRL levels decreased; SHBG, FSH, and APN levels increased; ovary volume level decreased; Rosenfield and hairy score levels decreased	NS	[50]
Liang et al., 2019	100	Kuntai capsule	Treatment arm: CHF Control arm: placebo	6 months	BMI and WHR levels decreased; FPG, 2hGLU, FINS, 2hINS, and HOMA-IR levels decreased; ISI level increased; TC, TG, and LDL-C levels decreased; HDL-C level improved; LH, LH/FSH, and T levels decreased	Mild adverse events (T: 3, C: 8); no SAE	[55]
Chen et al., 2017	114	Invigorating kidney and dispersing stagnated liver qi and promoting blood tablet	Chinese herbs arm: CHF Western medicine arm: metformin Combined arm: CHF + metformin	3 months	Cure (19) Moderately improved (18) Ineffective (2) Overall efficacy: 94.87% BMI, WHR, and central obesity rate levels decreased; TC, TG, and LDL-C levels decreased; HDL-C level increased	NS	[56]
Wei et al., 2014	124	Qiangshen table combined with Longlu capsule	Treatment arm: CHF Control arm: metformin	6 months	Markedly improved (48) Moderately improved (22) Ineffective (3) Overall efficacy: 95.89% BMI, FINS, HOMA-IR, and visfatin levels decreased	AEs: none	[57]
Lu et al., 2015	121	Bushen Huatan fang	Treatment arm: CHF Control arm: metformin	3 menstrual cycles	BMI, waist circumference, and hip circumference levels decreased; 1hGLU and HOMA levels decreased; CHO, LDL-C, and ApoB levels decreased; SHBG level increased; acanthosis nigricans scores level decreased	AEs: none	[58]
Chen et al., 2016	93	Quyu Huatan decoction	Treatment arm: CHF Control arm: metformin	3 menstrual cycles	Cure (10) Markedly improved (19) Moderately improved (13) Ineffective (5) Overall efficacy: 89.4% BMI level decreased; FPG and INS levels decreased; TG and TC levels decreased; HDL level increased; FSH, LH, and T levels decreased	NS	[59]
Deng et al., 2019	117	Dingkun pill	A arm: Diane-35 B arm: CHF C arm: CHF + Diane-35	3 months	1hGLU and HOMA-IR levels decreased; QUICKI level increased	AEs: none	[63]
Hua et al., 2003	107	Yishen Jianpi Yangxue Jianpi Tongli therapy fang	Treatment arm: CHF Control arm: clomiphene	6 months	Cure (39) Moderately improved (29) Ineffective (8) Overall efficacy: 89.5% Pregnancy rate level increased; BMI and F-G scores levels decreased; 1hGLU and 2hGLU levels decreased; LH and T levels decreased	AEs: none	[70]
Ma et al., 2017	147	Bushen Tiaochong decoction	Treatment arm: CHF + Diane-35 + letrozole Control arm: Diane-35 + letrozole	3 menstrual cycles	Markedly improved (31) Moderately improved (35) Ineffective (7) Overall efficacy: 90.41% Endometrial thickness level increased; BMI, LEP, and VF levels decreased; APN level increased; LH, FSH, and LH/FSH levels decreased; E_2_ level increased; PI and RI levels decreased	Nausea (T: 5, C: 4); headache (T: 0, C: 1); no SAE	[71]
Zhang, 2017	113	Tiaojing Huoxue decoction	Treatment arm: CHF + ethinylestradiol cyproterone acetate Control arm: ethinylestradiol cyproterone acetate	3 months	Markedly improved (31) Moderately improved (18) Ineffective (8) Overall efficacy: 85.96% HOMA-IR and LP levels decreased; T and LH levels decreased; TNF-*α* and IL-8 levels decreased; IGF-1 level increased	NS	[72]
Cao et al., 2014	105	Kuntai formula	Treatment arm: CHF Control arm: Diane-35	3 months	Markedly improved (21) Moderately improved (22) Ineffective (5) Overall efficacy: 89.6% Ovulation rate and pregnancy rate levels increased; BMI level decreased; LH, T, and leptin levels decreased; SHBG level increased	NS	[73]
Teng et al., 2019	227	YiShen JianPi decoction	A arm: lifestyle modification B arm: lifestyle modification + metformin C arm: lifestyle modification + CHF	3 months	Embryo implantation rate level increased; TC, TG, and LDL-C levels decreased; HDL-C level increased; WHR level increased; T level decreased (C arm)	NS	[74]
Wang, 2011	90	Shugan Tiaojing formula	Treatment arm: CHF Control arm: CHF + Diane-35 + clomiphene	3 menstrual cycles	Cure (6) Moderately improved (32) Ineffective (7) Overall efficacy: 84.4% FSH and E_2_ levels increased; LH, PRL, and T levels decreased; SDS and SAS score levels decreased	NS	[75]

**Table 2:** Summary of the effect of randomized studies of single herb on PCOS outcomes.

Authors (year)	Herbal medicine Scientific name	Extract and dosage form	Interventions	Study duration	Results	AEs	References
Jamilian et al., 2020	Rhizoma Curcumae	Curcumin capsule	Treatment arm: curcumin Control arm: placebo	12 weeks	BMI and weight levels decreased; FPG, FINS, and HOMA-IR levels decreased; QUICKI level increased; TC and LDL-C levels decreased; HDL-C level increased; PPAR-g and LDLR gene expression levels increased	NS	[84]
Wu et al., 2019	Rhizoma Curcumae	Curcuma water decoction	Treatment arm: curcuma + metformin Control arm: metformin	3 months	BMI and WHR levels decreased; TC, TG, and LDL-C levels decreased; HDL-C level increased; FAI and LH/FSH levels decreased; FPG, 2hGLU, FINS, 2hINS, HbA1c, and HOMA-IR levels decreased	Gastrointestinal complications, dizziness, pruritus, and edema (T: 10, C: 13)	[85]
Wang et al., 2011	Rhizoma Coptidis	Berberine tablet	Treatment arm: berberine + metformin Control arm: metformin	3 months	Ovulation rate level increased; BMI, FIN, and HOMA-IR levels decreased; LH, LH/FSH, and T levels decreased	NS	[108]
Kamel, 2013	Rhizoma Cimicifugae	Klimadynon	Treatment arm: klimadynon Control arm: clomiphene	3 months	LH and FSH/LH levels decreased; P level increased; endometrial thickness level increased	Hyperstimulation (T: 1, C: 2)	[109]
Bashtian et al., 2013	Semen Trigonellae	Fenugreek seed extract as capsule	Treatment arm: fenugreek + metformin Control arm: placebo + metformin	8 weeks	Menstrual cyclicity improved; polycystic-appearing ovaries decreased	AEs: none	[111]
Wang et al., 2016	Radix Salviae	Tanshinone capsule	Treatment arm: tanshinone + letrozole Control arm: placebo + letrozole	3 months	Ovulation rate and pregnancy rate levels increased; BMI level decreased; TC and TG levels decreased; LH, FSH, and T levels decreased	NS	[112]
Li et al., 2017	Rhizoma Coptidis	Berberine capsule	Treatment arm: berberine Control arm: metformin	3 months	BMI, HOMA-IR, FPG, FINS, and 2hINS levels decreased; TC, TG, and LDL-C levels decreased; LH, LH/FSH, and T levels decreased	Gastrointestinal complications (T: 0, C: 1)	[114]
Li et al., 2015	Rhizoma Coptidis	Berberine	Treatment arm: berberine + dydrogesterone Control arm: dydrogesterone	3 months	HOMA-IR level decreased; TC and TG levels decreased; LH, FSH, T, DHEA, and A levels decreased	NS	[115]
An et al., 2014	Rhizoma Coptidis	Berberine tablet	BBR arm: berberine MET arm: metformin Placebo arm: placebo	3 months	Live birth rate level increased; BMI and WHR levels decreased; TC and LDL-C levels decreased (BBR arm)	Nausea (MET: 12, BBR: 9, placebo: 4), OHSS (MET: 2, BBR: 2, Placebo: 6), no SAE	[116]
Wu et al., 2016	Rhizoma Coptidis	Berberine capsule	Letrozole arm: letrozole + berberine placebo Berberine arm: berberine + letrozole placebo Combination arm: letrozole + berberine	6 months	Ovulation rate level mild Increased (combination arm); BMI and waist circumference levels decreased (berberine arm)	Mild in three arms: gastrointestinal complications in three groups; no SAE	[117]
Kort et al., 2014	Cortex Cinnamomi	Cinnamon capsule	Treatment arm: cinnamon Control arm: placebo	6 months	Menstrual cyclicity improved	NS	[118]
Sohaei et al., 2019	Rhizoma Curcumae	Curcumin	Treatment arm: curcumin + metformin Control arm: placebo + metformin	6 weeks	FINS and HOMA-IR levels decreased; QUICKI level increased	Gastrointestinal complications (T: 3, C: 0)	[124]
Heshmati et al., 2020	Rhizoma Curcumae	Curcumin capsule	Treatment: curcumin Control arm: placebo	3 months	GPx level increased; SIRT1 and PGC-1a gene expression levels increased	AEs: none	[125]
Su et al., 2015	Radix Salviae	Tanshinone capsule	Treatment arm: tanshinone Control arm: placebo	3 months	1hGLU and 2hGLU levels decreased; serum glucose clearance rate level increased	NS	[126]
Zhang et al., 2015	Radix Salviae	Tanshinone capsule	Treatment arm: tanshinone Control arm: placebo	3 months	TC and TG levels decreased; HDL-C level increased; T level decreased	AEs: none	[127]
Wu et al., 2016	Radix Salviae	Tanshinone capsule	Treatment arm: tanshinone + Diane-35 Control arm: Diane-35	3 months	TC, TG, and LDL-C levels decreased; HDL-C level increased; LH and TSH levels decreased; GH level increased; ACTH, *β*-EP, Cor, and UFC levels decreased	NS	[128]
Li et al., 2017	Radix Puerariae	Puerarin tablet	Obesity treatment arm: puerarin + Diane-35 + metformin Thin treatment arm: puerarin + Diane-35 + metformin Obesity control arm: Diane-35 + metformin	3 months	Menstrual cyclicity improved; BMI level decreased; HOMA-IS level decreased; TC and TG levels decreased; HDL-C level increased; SOD level increased; T level decreased; (obesity treatment arm)	NS	[131]
Khan et al., 2018	Cortex Cinnamomi	Darchini capsule	Treatment arm: darchini Control arm: metformin	60 days	Menstrual cyclicity improved; ovarian size level decreased; FT level decreased	Epigastric burning and belching (T: 1, C: 1); no SAE	[132]
Wang et al., 2007	Cortex Cinnamomi	Cinnamon extract as capsule	Treatment arm: cinnamon Control arm: placebo	8 weeks	FPG and HOMA-IR levels decreased; QUICKI and matsuda insulin resistance index levels increased	NS	[136]

**Table 3:** Summary of the effect of randomized studies of dietetic therapy on PCOS outcomes.

Authors (year)	Design	Form and composition	Interventions	Duration of study	Results	References
Liu et al., 2017	RCT	POT: Radix Astragali seu Hedysari + Rhizoma Polygonati + Rhizoma Dioscoreae + Herba Dendrobii + Radix Morindae Officinalis + equal	Treatment arm: oral warm nest pot + letrozole Control arm: letrozole	3 months	Markedly improved (24) Moderately improved (8) Ineffective (5) Overall efficacy: 87.18% Ovulation rate level increased; menstrual cyclicity improved	[156]
Liu et al., 2019	RCT	Porridge and Tea: (1) Poria Rhizoma Dioscoreae Porridge: Poria Rhizoma + Dioscoreae + rice (2) Pericarpium Citri Reticulatae Fructus Hordei Germinatus Porridge: Pericarpium Citri Reticulatae + Fructus Hordei Germinatus + rice (3) Fructus Lycii Rhizoma Dioscoreae Porridge: Fructus Lycii + Rhizoma Dioscoreae + rice (4) Fructus Citri Sarcodactylis Pericarpium Citri Reticulatae tea: Fructus Citri Sarcodactylis + Pericarpium Citri Reticulatae + tea (5) Flos Rosae Rugosae Fructus Citri Sarcodactylis tea: Flos Rosae Rugosae + Fructus Citri Sarcodactylis + tea	Treatment arm: dietotherapy + acupoint embedding therapy Control arm: acupoint embedding therapy	3 months	Markedly improved (17) Moderately improved (22) Ineffective (4) Overall efficacy: 90.7%	[157]
Tehrani et al., 2017	RCT/double-blind	Tea: green tea	Treatment arm: tea Control arm: placebo	12 weeks	Weight and fasting insulin levels decreased; FT levels decreased	[159]
Farhadian et al., 2020	RCT/double-blind	Tea: green tea	Green tea arm: tea Metformin arm: metformin Control arm: placebo	3 months	BMI, weight, waist circumference, and hip circumference levels decreased	[160]
Mombaini et al., 2017	RCT/double-blind	Tea: green tea	Treatment arm: tea Control arm: placebo	45 days	BMI, weight, body fat, waist circumference, and hip circumference levels decreased	[161]
Grant, 2010	RCT/double-blind	Tea: spearmint tea	Treatment arm: tea Control arm: placebo herbal tea	1 month	FT and TT levels decreased; FSH and LH levels increased; hirsutism scored level decreased	[162]
Haj-Husein et al., 2016	RCT/double-blind	Tea: marjoram tea	Treatment arm: tea Control arm: placebo	1 month	HOMA-IR level decreased; DHEA-S level decreased	[163]
Chan et al., 2006	RCT/double-blind	Tea: Lung Chen tea	Treatment arm: tea Control arm: placebo	3 months	Weight level decreased; TC level decreased	[164]
